# Three-dimensional growth of tibial shaft ossification in the human fetus: a digital-image and statistical analysis

**DOI:** 10.1007/s00276-018-2138-6

**Published:** 2018-11-23

**Authors:** Mariusz Baumgart, Marcin Wiśniewski, Magdalena Grzonkowska, Mateusz Badura, Michał Szpinda, Katarzyna Pawlak-Osińska

**Affiliations:** 1 Department of Normal Anatomy, The Ludwik Rydygier Collegium Medicum in Bydgoszcz, The Nicolaus Copernicus University in Toruń, Łukasiewicza 1 Street, 85-821, Bydgoszcz, Toruń Poland; 2 Department of Otolaryngology and Oncology, The Ludwik Rydygier Collegium Medicum in Bydgoszcz, The Nicolaus Copernicus University in Toruń, Toruń, Poland

**Keywords:** Tibia, Bone development, Osteogenesis, Fetal development

## Abstract

**Purposes:**

Tibial shaft ossification in terms of its size and growth may be criticalin describing both the fetal stage and maturity, and in identifying innate disorders. The present study was executed to quantitatively assess ossification of the tibial shaft, taking its morphometric linear, planar and volumetric parameters into account.

**Materials and methods:**

With the use of methods of CT, digital-image analysis and statistics, the evolutionof tibial shaft ossification in 47 spontaneously aborted human fetuses at the age of 17–30 weeks was studied.

**Results:**

Without any male–female and right-left morphometric differences, the best fit growth dynamics fortibial shaft ossification was modelled by the following functions: *y* = 5.312 + 0.034 × (age)^2^ ± 0.001 (*R*^2^ = 0.89) for its length, *y* = − 2.855 + 0.307 × age ± 0.009 (*R*^2^ = 0.96) for its proximal transverse diameter, *y* = − 0.758 + 0.153 × age ± 0.005 (*R*^2^ = 0.88) for its middle transverse diameter, *y* = − 1.844 + 0.272 × age ± 0.09 (*R*^2^ = 0.90) for its distal transverse diameter, *y* = − 40.263 + 0.258 × (age)^2^ ± 0.007 (*R*^2^ = 0.94) for its projection surface area, and *y* = − 287.996 + 1.186 × (age)^2^ ± 0.037 (*R*^2^ = 0.92) for its volume. The femoral–to–tibial ossification length ratio was 1.15 ± 0.1.

**Conclusions:**

The size of tibial shaft ossification displays neither sex nor laterality differences. Tibial shaft ossification follows quadratic functions with respect to its length, projection surface area and volume, and linear functions with respect to its proximal, middle and distal transverse diameters. The obtained morphometric data of tibial shaft ossification are considered normative age-specific references of relevance in both the estimation of fetal ages and the ultrasound diagnostics of congenital defects.

## Introduction

A typical feature of newborns delivered at term are ossified shafts of long bones. Therefore, starting with the 2nd trimester of pregnancy, routine ultrasound measurements of limb ossified shafts are conducted so as to assess the development of the fetus and gestational age. Precisely, with the use of ultrasound, measuring long bones is adequate from week 12 of fetal life [1]. Moreover, ultrasonic examinations allow for either detection or elimination of skeletal dysplasias [2]. The effectiveness of ultrasound ranges from 40 to 60%, thus using ultrasound alone is insufficient to provide a comprehensive diagnosis. Therefore, diagnostic imaging with the use of radiographic and computed tomography techniques is extremely conducive [3, 4]. Compared to 2D X-ray and USG, CT examinations eliminate an overlap of anatomical structures and allow for an easy distinction between different body tissues. Furthermore, a big advantage of a CT technique is the possibility of observing an examined structure in any plane and at any time without sacrificing image details [5]. In addition, the length of fetal limbs can be used in establishing the time of intrauterine death [6]. Although the most common bony measurements refer to the femur and humerus, then extensive diagnostics with the evaluation of other long bones is indispensable, when any fetal malformations are suspected [7]. Due to their increased mobility, two bones in the forearm or leg are usually measured conjointly, without distinguishing separate bones, that are the ulna and radius, and the tibia and fibula [1, 6, 8]. Of those two sets of bones, it is much easier to measure the tibia and fibula because of their relatively fixed position [1].

We failed to find any reports in the medical literature concerning dimensions of tibial shaft ossification.

Thus, in the present study we aimed:

- to perform morphometric analysis of tibial shaft ossification in human fetuses (linear, superficial and spatial parameters) to determine their normative values,
- to establish possible differences between sexes for all analyzed parameters; and
- to compute growth dynamics for the analyzed parameters, expressed by best-matched mathematical models.

## Materials and methods

The study material was consisted of 47 human fetuses of both sexes (25 males and 22 females) aged 17–30 weeks of fetal life, derived from spontaneous abortions or preterm deliveries. The material was acquired before the year 2000 and remains part of the specimen collection of the Department of Normal Anatomy of our university. The study was approved by Bioethics Committee of our university (KB 275/2011). The fetal ages were determined on the base of crump–rump length. Table 1 lists the characteristics of the study group, including age, number and sex of the fetuses.

**Table 1:** Age, number and sex of fetuses

Gestational age (weeks)	Crown-rump length (mm)				Number of fetuses	Sex	
	Mean	SD	Min.	Max.		♂	♀
17	116.00	1.41	115.00	117.00	2	1	1
18	130.00	0.00	130.00	130.00	2	1	1
19	150.00	3.03	146.00	154.00	6	3	3
20	159.50	0.71	159.00	160.00	2	1	1
21	174.75	2.87	171.00	178.00	4	3	1
22	184.67	1.53	183.00	186.00	3	1	2
23	197.75	2.99	195.00	202.00	4	3	1
24	208.57	3.74	204.00	213.00	7	4	3
25	214.50	0.71	214.00	215.00	2	1	1
26	226.00	1.41	225.00	227.00	2	1	1
27	237.75	2.75	235.00	241.00	4	3	1
28	246.67	4.93	241.00	250.00	3	1	2
29	254.00	1.41	253.00	255.00	2	1	1
30	263.25	1.26	262.00	265.00	4	1	3
Total					47	25	22

**Fig. 1:**
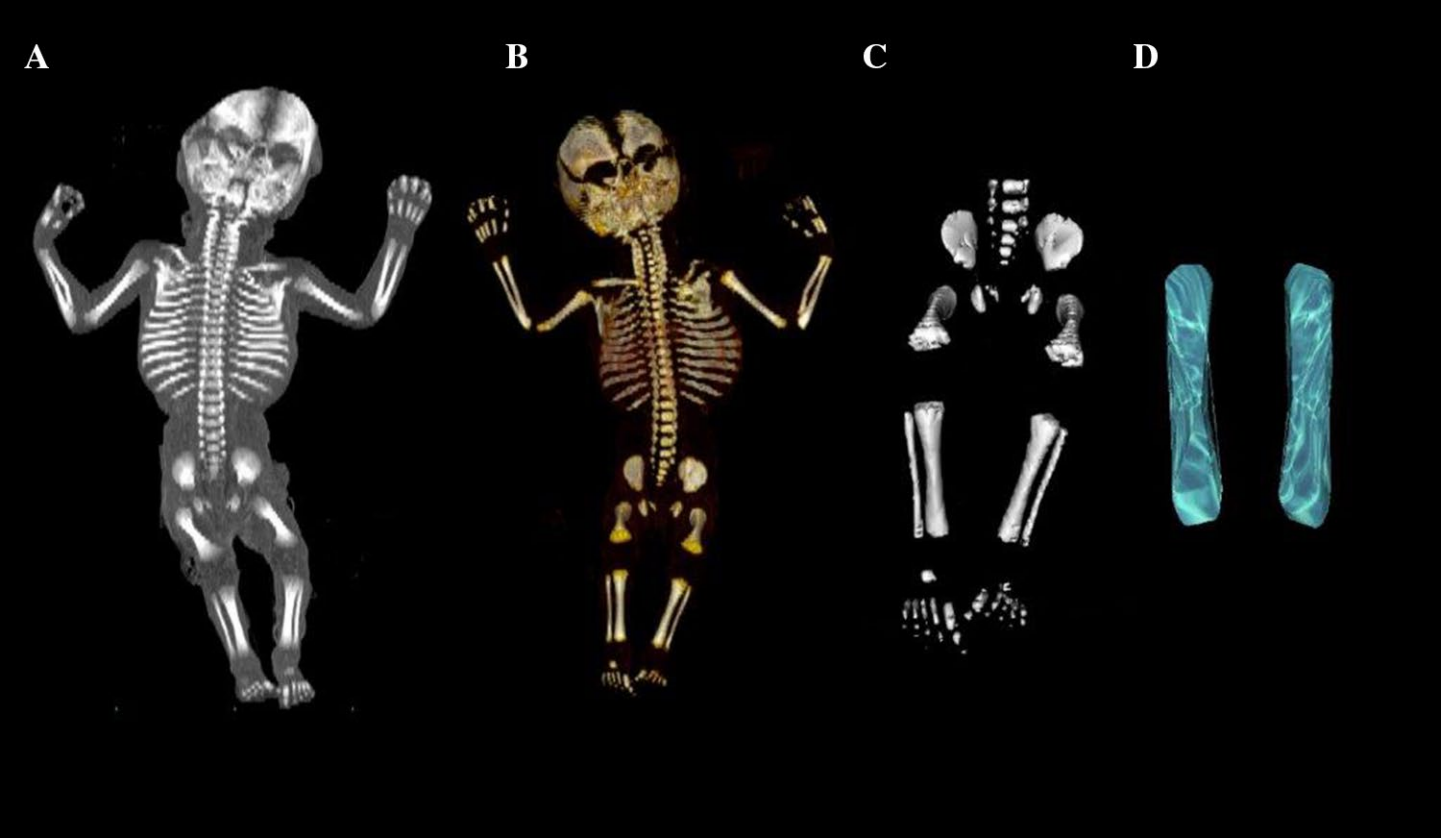
CT of a male fetus aged 19 weeks (in the frontal projection) recorded in DICOM formats (**a**), with the frontal reconstruction of fetal bones (**b**), with the frontal projection of the fetal pelvic girdle, and right and left lower limbs (**c**), with 3D reconstructions of ossification in the right and left tibial shafts (**d**) of the fetus, assessed by Osirix 3.9

Using the Siemens–Biograph 128 mCT scanner, scans of fetuses in DICOM formats were acquired at 0.4 mm intervals (Fig. 1). Despite the cartilaginous stage, contours of the proximal and distal ends of tibial shaft ossification were already clearly visible [9, 10], thus enabling us to perform its morphometric analysis with relation to its length, transverse and sagittal dimensions, and volume.

Using a Siemens-Biograph 128 mCT camera (Siemens Healthcare GmbH, Erlangen, Germany) placed at Department of Positron Emission Tomography and Molecular Imaging (Oncology Center, Collegium Medicum of the Nicolaus Copernicus University, Bydgoszcz, Poland), the fetuses were scanned at a step of 0.4 mm, recorded in DICOM formats (Fig. 1), and subsequently subjected to morphometric analysis with the use of the Osirix 3.9 software. It should be emphasized that Osirix 3.9 permits precise numerical analysis of any type of linear, planar and three-dimensional reconstructions of the studied objects.

The gray scale in Hounsfield units of achieved CT pictures ranged from − 275 to − 134 for a minimum, and from + 1165 to + 1558 for a maximum. Thus, the window width (WW) alternated from 1.404 to 1.692, and the window level (WL) varied from + 463 to + 712. The details of the imaging protocol were: mAs—60, kV—80, pitch—0.35, FoV—180, rot. time—0.5 s, while the details of CT data were: slice thickness 0.4 mm, image increment 0.6 mm, and kernel—B45 f-medium. Of note, both WW and WL optimize the appearance of CT images by determining the contrast and brightness levels assigned to the CT image data. WW directly refers to the maximal number of shades of grey to be displayed on a CT monitor, and expressed by the range of HU. WL is referred to as the midpoint of the range of the CT numbers displayed (window center).

**Fig. 2:**
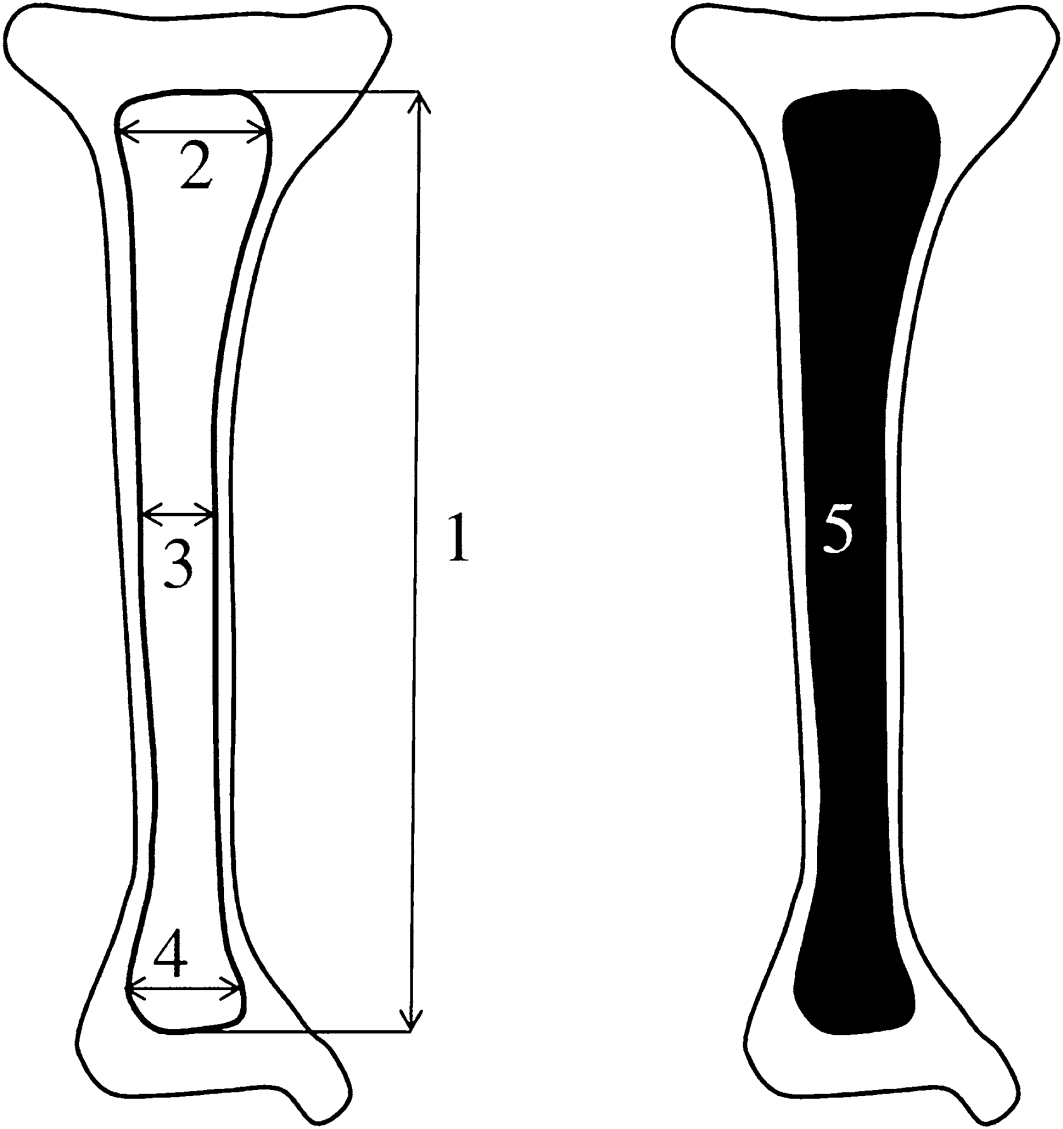
Diagram showing measurements of tibial shaft ossification in the horizontal projection: (1) length, (2) proximal transverse diameter, (3) middle transverse diameter, (4) distal transverse diameter, (5) projection surface area

Measurements of tibial shaft ossification were conducted in a specific order (Fig. 2). In each fetus, the assessment of linear dimensions, projection surface area and volume of tibial shaft ossification was carried out. The bilateral quantitative evaluation of six parameters of tibial shaft ossification was conducted, including:

1. length, based on the determined distance between the proximal and distal borderlines of tibial shaft ossification in the frontal plane (Fig. 2);
2. proximal transverse diameter, based on the determined distance between the medial and lateral borderlines of the proximal region of tibial shaft ossification in the frontal plane (Fig. 2);
3. middle transverse diameter, based on the determined distance between the medial and lateral borderlines of the central region of tibial shaft ossification in the frontal plane (Fig. 2);
4. distal transverse diameter, based on the determined distance between the medial and lateral borderlines of the distal region of tibial shaft ossification in the frontal plane (Fig. 2);
5. projection surface area, based on the determined contour of tibial shaft ossification in the frontal plane (Fig. 2);
6. volume, calculated using advanced diagnostic imaging tools for 3D reconstruction, taking into account the position and absorption of radiation by bone tissue (Fig. 1d).

All measurements were performed by one researcher (MB). Each measurement was performed three times under the same conditions but at different times, and averaged. The numerical data obtained were statistically analyzed. Distribution of variables was checked using the Shapiro–Wilk (W) test, while homogeneity of variance was checked using Fisher’s test. The results were expressed as arithmetic means with standard deviations (SD). To compare the means, the Student *t* test for independent variables and one-way analysis of variance were used. Tukey’s test was used for post hoc analysis. If no similarity of variance occurred, the non-parametric Kruskal–Wallis test was used. The characterization of developmental dynamics of the examined parameters was based on linear and curvilinear regression analysis. The match between the numerical data and computed regression curves was evaluated based on the coefficient of determination (*R*^2^).

## Results

The statistical analysis revealed neither significant sex nor bilateral differences, which allowed us to compute only one growth curve for each analyzed parameter. Mean values and standard deviations of the analyzed parameters of tibial shaft ossification on the right and left sides in human fetuses at the analyzed gestational stages are presented in Tables 2 and 3 for length and proximal, middle and distal transverse diameters, and in Table 4 for projection surface area and volume.

**Table 2:** Length and transverse diameters for: proximal end, middle part and distal end of right tibial shaft ossification in human fetuses

Gestational age (weeks)	Number of fetuses	Right tibial shaft ossification							
		Length (mm)		Transverse diameter (mm)					
				Proximal end		Middle part		Distal end	
		Mean	SD	Mean	SD	Mean	SD	Mean	SD
17	2	14.45	0.84	2.39	0.03	1.82	0.06	2.79	0.06
18	2	16.27	0.36	2.63	0.08	1.87	0.01	2.93	0.01
19	6	18.30	1.46	2.91	0.12	2.09	0.12	3.23	0.10
20	2	22.82	1.83	3.37	0.10	2.44	0.07	3.51	0.11
21	4	20.54	2.36	3.68	0.14	2.66	0.19	4.16	0.18
22	3	20.73	0.55	4.13	0.21	2.60	0.37	4.22	0.60
23	4	23.39	1.73	3.79	0.36	2.43	0.21	4.23	0.41
24	7	26.56	3.31	4.81	0.38	3.14	0.23	4.96	0.45
25	2	26.69	1.22	5.24	0.07	3.25	0.07	4.67	0.07
26	2	27.08	0.91	5.06	0.17	3.26	0.18	5.82	0.10
27	4	29.51	1.27	5.21	0.11	3.33	0.17	5.28	0.18
28	3	31.61	0.61	5.59	0.11	3.59	0.14	5.62	0.07
29	2	33.13	0.20	5.90	0.10	3.81	0.07	5.93	0.28
30	4	35.41	2.01	6.40	0.21	4.10	0.07	6.51	0.24

**Table 3:** Length and transverse diameters for: proximal end, middle part and distal end of left tibial shaft ossification in human fetuses

Gestational age (weeks)	Number of fetuses	Left tibial shaft ossification							
		Length (mm)		Transverse diameter (mm)					
				Proximal end		Middle part		Distal end	
		Mean	SD	Mean	SD	Mean	SD	Mean	SD
17	2	14.11	0.43	2.25	0.20	1.90	0.06	2.73	0.12
18	2	14.58	0.24	2.66	0.00	2.06	0.03	2.91	0.09
19	6	16.97	1.43	2.98	0.33	2.22	0.05	3.25	0.19
20	2	20.70	1.23	3.54	0.15	2.32	0.01	3.57	0.31
21	4	19.52	2.06	3.68	0.60	2.68	0.22	3.87	0.29
22	3	22.38	1.71	3.51	0.32	2.49	0.26	4.43	0.12
23	4	24.23	3.45	3.76	0.31	2.65	0.09	4.36	0.69
24	7	26.31	3.43	4.83	0.57	2.92	0.24	4.86	0.52
25	2	27.03	1.22	5.24	0.23	3.35	0.09	4.62	0.13
26	2	26.94	1.36	5.19	0.02	2.93	0.06	5.19	0.66
27	4	29.91	1.25	5.47	0.13	3.15	0.05	5.31	0.23
28	3	31.82	0.26	5.79	0.11	3.50	0.02	5.58	0.04
29	2	32.35	0.14	6.01	0.04	3.57	0.01	5.90	0.13
30	4	35.52	2.48	6.32	0.23	3.84	0.14	6.37	0.19

**Table 4:** Projection surface area and volume of tibial shaft ossification

Gestational age	Number of fetuses	Tibial shaft ossification							
		Projection surface area (mm^2^)				Volume (mm^3^)			
		Right tibia		Left tibia		Right tibia		Left tibia	
		Mean	SD	Mean	SD	Mean	SD	Mean	SD
17	2	33.62	0.32	35.69	1.97	83.48	17.88	80.01	17.56
18	2	38.61	1.27	38.75	0.57	101.88	0.13	100.49	4.90
19	6	52.50	7.39	53.28	9.74	141.42	17.29	138.14	14.70
20	2	68.81	7.95	73.35	11.84	241.65	37.67	183.21	29.87
21	4	73.01	21.82	67.74	19.97	255.17	89.23	245.10	28.32
22	3	82.90	12.52	86.24	1.44	219.27	41.46	291.15	18.45
23	4	88.24	18.33	96.14	9.63	305.07	46.41	325.00	14.42
24	7	103.79	15.38	120.40	21.70	430.77	126.57	403.12	92.80
25	2	135.98	3.26	104.55	1.82	395.86	6.08	496.36	150.16
26	2	140.87	0.60	143.10	1.22	430.04	2.49	418.65	12.16
27	4	147.83	2.75	144.83	7.46	556.23	44.26	553.11	63.51
28	3	156.72	5.33	163.64	8.77	621.76	11.13	624.28	1.59
29	2	172.68	2.58	174.75	0.85	674.32	0.62	713.66	49.77
30	4	194.20	14.91	190.63	5.75	823.91	74.68	848.16	70.29

**Fig. 3:**
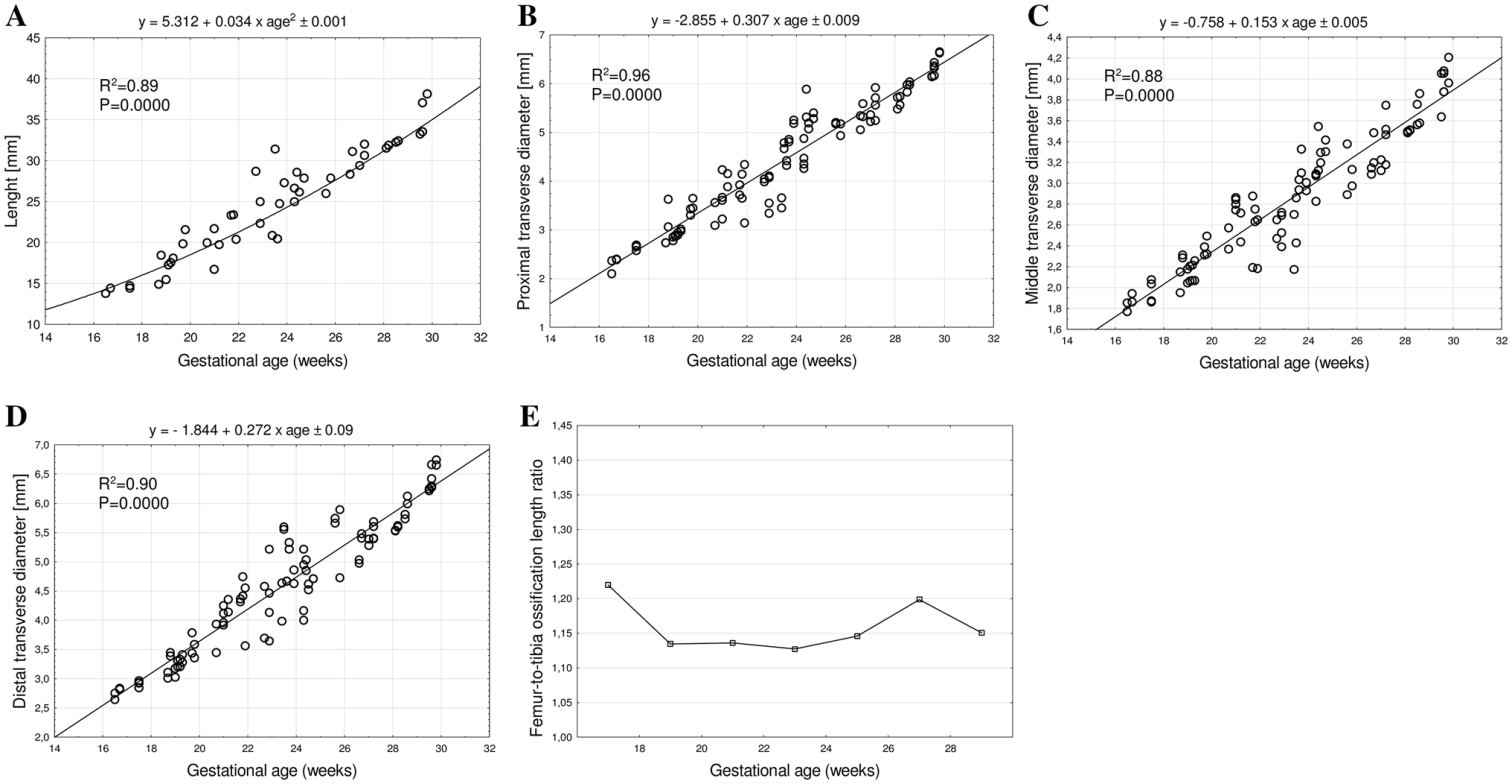
Regression lines for length (**a**), proximal (**b**), middle (**c**) and distal (**d**) transverse diameters, and femur-to-tibia length ossification ratio (**e**) of tibial shaft ossification

In fetuses between 17 and 30 weeks of gestation, the mean length of tibial shaft ossification increased from 14.45 ± 0.84 to 35.41 ± 2.01 mm on the right side, and from 14.11 ± 0.43 to 35.52 ± 2.48 mm on the left side, following the quadratic function: *y* = 5.312 + 0.034 × (age)^2^ ± 0.001 (*R*^2^ = 0.89), as presented in Fig. 3a.

The mean proximal transverse diameter of tibial shaft ossification ranged from 2.39 ± 0.03 mm at 17 weeks to 6.4 ± 0.21 mm at 30 weeks on the right side, and from 2.25 ± 0.20 to 6.32 ± 0.23 mm on the left side, according to the linear function: *y* = − 2.855 + 0.307 × age ± 0.009 (*R*^2^ = 0.96), as displayed in Fig. 3b. At the fetal ages of 17–30 weeks, the mean middle transverse diameter of the tibial shaft ossification ranged from 1.82 ± 0.06 to 4.10 ± 0.07 mm on the right side, and from 2.25 ± 0.20 to 6.32 ± 0.23 mm on the left side, following the linear function: *y* = − 0.758 + 0.153 × age ± 0.005 (*R*^2^ = 0.88)—(Fig. 3c). At that time, the mean distal transverse diameter of tibial shaft ossification ranged from 2.79 ± 0.06 to 6.51 ± 0.24 mm on the right side, and from 2.73 ± 0.12 to 6.37 ± 0.19 mm on the left side, following the linear function: *y* = − 1.844 + 0.272 × age ± 0.09 (*R*^2^ = 0.90)—(Fig. 3d).

Based on our previous numerical data regarding the length of femoral shaft ossification [11], the femur-to-tibia ossification length ratio was calculated as a quotient of the lengths of ossification of the femoral and tibial shafts. The femur-to-tibia ossification length ratio in the analyzed period between 17 and 30 weeks of gestation was 1.15 ± 0.1 (Fig. 3e).

**Fig. 4:**
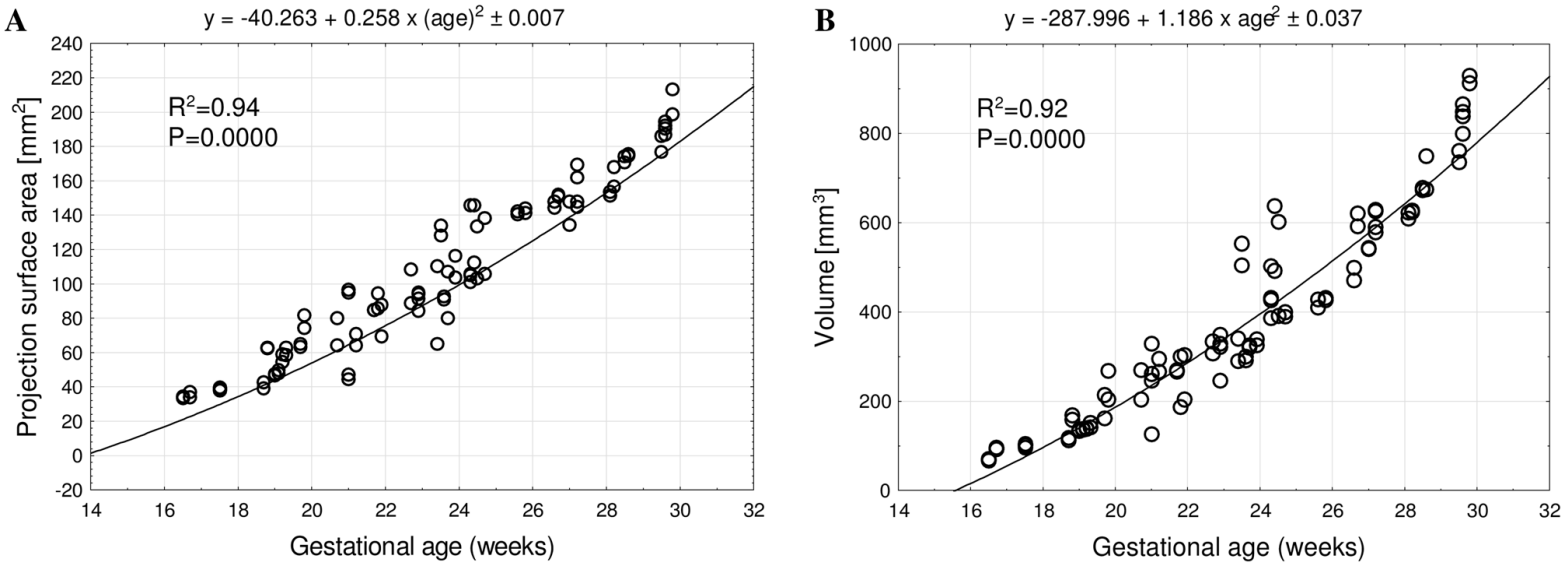
Regression lines for projection surface area (**a**) and volume (**b**) of tibial shaft ossification

The mean projection surface area of tibial shaft ossification ranged from 33.62 ± 0.32 mm^2^ at 17 weeks to 94.20 ± 14.91 mm^2^ at 30 weeks on the right side, and from 35.69 ± 1.97 to 190.63 ± 5.75 mm^2^, respectively, on the left side, following the quadratic function: *y* = − 40.263 + 0.258 × (age)^2^ ± 0.007 (*R*^2^ = 0.94)—(Fig. 4a).

The mean volume of tibial shaft ossification in the fetal age range of 17–30 weeks increased from 83.48 ± 17.88 to 823.91 ± 74.68 mm^3^ on the right side, and from 80.01 ± 17.56 to 848.16 ± 70.29 mm^3^ on the left side, following the quadratic function of age: *y* = − 287.996 + 1.186 × (age)^2^ ± 0.037 (*R*^2^ = 0.92)—(Fig. 4b).

## Discussion

The development of the lower limb includes transformation of the cartilaginous skeleton into osseous one at the three consecutive stages, i.e. the ossification process first involves the shafts of long bones, followed by their epiphyses and, finally encompasses osseous processes. Lower limb ossification commences as early as at week 7 of fetal life and involves the femur, followed by the tibia [11–14].

Tibial ossification originates at the tibial mid-shaft and advances towards the proximal and distal endings. The ossification process of the femoral shaft has a different course than that of the femoral epiphyses. The primary ossification center appears in the mid-shaft at approx. week 8 of fetal life, while the secondary ossification centers located within the proximal and distal epiphyses appear starting with the 3rd trimester of pregnancy [12].

After reviewing the medical literature in the fields of radiology, gynecology, obstetrics, pediatrics, forensic medicine judicial and orthopedics, X-ray and ultrasound imaging proved to be the main methods to examine the development and dimensions of the lower limb ossification centers. In this aspect, we did not manage to find any reports of the use of CT in studies on ossification in fetal lower limbs.

Using X-ray, studies about ossification of lower limb bone epiphyses, including the proximal epiphysis of the tibia, were already precisely described. Pryse-Davies et al. [15] examined 379 autopsied fetuses at the age of 21–42 weeks and found a faster development of ossification in female fetuses. These authors also demonstrated that in fetuses with lethal malformations, the growth of ossification was either significantly retarded or accelerated. A clearly slower development of ossification was observed in fetuses with low birth weight associated with D-trisomy or E-trisomy, lethal dysplasia, as well as primary developmental defects of long bones. In turn, an accelerated growth of ossification occurred in fetuses with anencephaly. In our study, tibial shaft ossification demonstrated neither sex nor laterality differences, which clearly corresponded with studies conducted using CT for femoral [11] and iliac [3] ossification in the human fetus. Khan and Faruqi [4] measured the shafts of long bones in X-ray examinations of 34 autopsied fetuses, previously fixed in 10% formalin solution. The tibia revealed an increase in length from 3.5 mm at week 3 to 68.5 mm at week 10 of gestation. The greatest monthly increase in tibial shaft length (30.5 mm) occurred during month 5, with the mean tibial length of 46.0 mm. The authors observed the length of tibial shaft to increase according to a natural logarithmic model.

Compared to radiological studies, one definite advantage of ultrasound imaging is the elimination of fetal exposure to X-rays and greater accuracy of findings, as measuring ossification using ultrasound is feasible starting with the object’s size of 1 mm, in contrast to 3 mm for radiographic imaging [16].

Basing on ultrasound examinations of 228 human fetuses aged 28–40 weeks, Goldstein et al. [17] measured ossification of the tibial proximal epiphysis. They found no proximal epiphysis of the tibia in fetuses aged up to week 34. However, at week 35 the proximal tibial epiphysis was distinguished in 35%, at week 37 it was present in 79%, while from the age of 39 weeks it was present in all fetuses. In 77% of fetuses under the age of 37 weeks, the size of the proximal tibial epiphysis ranged from 1 to 2 mm, while in 94% of fetuses over the age of 38 weeks, its size was equal to or greater than 3 mm. According to these authors, the ossification process of the proximal tibial epiphysis has a specific developmental pattern that is noticeable in ultrasound examination and provides additional information about the fetal age in the 3rd trimester of pregnancy. In turn, Donne et al. [16] demonstrated the presence of ossification of the proximal tibial epiphysis in one of 35 fetuses aged 32 weeks, in 17% of fetuses aged 34 weeks, in 33% of fetuses aged 36 weeks, in 83% of fetuses aged 37 weeks, in 97% of fetuses aged 39 weeks, and in all fetuses aged 40 weeks. Using ultrasound examination of 84 fetuses, Chinn et al. [18] observed the ossification process of the proximal tibial epiphysis to commence with week 35 of gestation. De Biasio et al. [19] ultrasonically measured the tibial length in 593 fetuses aged 10–14 weeks. An increase in tibial length followed the quadratic function: *y* = 0.0002 (age)^2^ + 0.0904 age − 0.3032, (SD = 0.0087 age + 0.4792). In turn, Chitty et al. [20] ultrasonically measured the tibial length in 663 fetuses aged 12–42 weeks. At that time, the tibial length for the 50th percentile increased from 7.6 ± 1.7 to 67.4 ± 3.2 mm. In terms of growth dynamics, the tibial length followed the function: *y* = 14451/(age)^2^ − 2553.2/age + 120.05, (SD = 0.049978 age + 1.1102. With the use of ultrasound, Exacoustos et al. [1] measured the lengths of some long bones, including the tibia, in 2175 fetuses aged 13–40 weeks. These authors found the growth of all measured bones to be directly proportionate to fetal age between weeks 13 and 28 of fetal life, while after week 28 of fetal life, the growth followed a quadratic function of age. Moreover, in the analyzed age range, the mean length of the tibia was 53.81 ± 10.56 mm, increasing from 16 mm at 15 weeks to 64 mm at 42 weeks of gestation. The tibia revealed an increase in length 2.23 ± 1.07 mm per week at the age range of 13–28 weeks and 1.54 ± 0.81 mm per week beyond week 28, in accordance with the function: *y* = − 32.294 + 3.739 age – 0.033 (age)^2^, (SD 1.619). In the present study involving fetuses aged 17–30 weeks, the length of tibial shaft ossification increased from 14.45 ± 0.84 to 35.41 ± 2.01 mm on the right side, and from 14.11 ± 0.43 to 35.52 ± 2.48 mm on the left side. Brons et al. [8] ultrasonically measured the length of the tibia in 63 fetuses aged 12–40 weeks and found the tibial length to increase from 4 to 66 mm, following a logarithmic function. These authors also calculated the femur-to-leg bone length ratio and the tibia-to-fibula length ratio, which were, respectively, 1.17 and 1.05 for week 12, 1.16 and 1.05 for week 16, 1.11 and 1.02 for week 20, 1.10 and 1.03 for week 24, 1.12 and 1.04 for week 28, 1.10 and 1.04 for week 32, 1.09 and 1.04 for week 36, and 1.08 and 1.04 for week 40 of fetal life. In the present study, we calculated the femoral-to-tibial ossification length ratio as 1.15 ± 0.1. With ultrasound, Zorzoli et al. [21] measured the lengths of long bones in 179 fetuses aged 64–108 days after the last menstrual period. These authors did not distinguish the tibia and fibula in their measurements, and reported just aggregate results for both bones. The length of the crural bones increased in a directly proportionate manner to fetal age, as *y* = − 19.633 + 0.31473 age. The mean tibia-to-fibula length reached the value of 0.99 ± 0.12. Reece et al. [22] used ultrasound examination to measure lengths of long bones, including the tibia, in fetuses from twin pregnancies and showed tibial length to increase in accordance with the quadratic function: *y* = − 22.4481 + 2.9063 × age − 0.01806 × (age)^2^. The authors did not find significant differences in length of crural bones between twin and singleton pregnancies. Based on ultrasound examinations of 530 human fetuses aged 13–42 weeks, Merz et al. [6] measured the length of the ossified tibial shaft, which increased from 9.0 ± 0.2 to 68.0 ± 0.5 mm.

This paper is the first account about the morphometric analysis of tibial shaft ossification in human fetuses with mathematical growth models. Table 5 includes growth dynamics for all examined parameters. The mean length, projection surface area and volume of tibial shaft ossification increased according to quadratic functions of age expressed in weeks. In turn, its proximal, middle and distal transverse diameters increased proportionately to age. It should be noted that the growth dynamics of the longitudinal growth of the femoral shaft ossification, as its increase in projection surface area, followed the quadratic function of fetal age *y* = 5.717 + 0.040 × (age)^2^ ± 2.905. Moreover, femoral ossification increased in transverse diameter in a proportionate pattern to fetal age: *y* = − 3.579 + 0.368 × age ± 0.529 for proximal diameter; *y* = − 1.105 + 0.187 × age ± 0.309 for middle diameter, and *y* = − 2.321 + 0.323 × age ± 0.558 for distal diameter. The volumetric growth of the femoral ossification increased following the cubic function: *y* = − 91.458 + 0.390 × (age)^3^ ± 92.146 [11]. A lack of reports concerning the size of tibial shaft ossification clearly precludes a more comprehensive discussion on this topic.

**Table 5:** Growth dynamics for length, proximal end, middle part, distal end, projection surface area and volume of tibial shaft ossification in human fetuses

Parameters	Functions
Tibial shaft ossification	
Length	*y* = 5.312 + 0.034 × (age)^2^ ± 0.001
Proximal transverse diameter	*y* = − 2.855 + 0.307 × age ± 0.009
Middle transverse diameter	*y* = − 0.758 + 0.153 × age ± 0.005
Distal transverse diameter	*y* = − 1.844 + 0.272 × age ± 0.09
Projection surface area	*y* = − 40.263 + 0.258 × (age)^2^ ± 0.007
Volume	*y* = − 287.996 + 1.186 × (age)^2^ ± 0.037

To date, more than 200 skeletal dysplasias with incidences from 2.3 to 7.6 per 10,000 births have been described. Among these skeletal defects, 51% are lethal dysplasias, which means 9 per 1000 prenatal deaths [23]. Skeletodysplasias are characterized by abnormal growth, development, differentiation, and consequently a deformed structure of bone and cartilage. One of such diseases is achondroplasia (short limbs), with the degree of long bone shortening classified as mild, moderate and severe [24]. According to Gonclaves and Jeanty [23], achondroplasia exerts the greatest effect on the length of long bones, dimensions of which are decreased by 40%. Furthermore, pathological changes in long bones may also be accompanied by pathological changes in the spine, exemplified by spondylodysplasia. A congenital disorder of the tibia can be manifested in two ways, mostly by a shortening of the tibia, which is associated with aplasia or dysplasia of the fibula. Much less frequent is congenital absence of the tibia, with the fibula developing normally [25–27]. Indubitably, the foremost examination in the assessment of the fetal growth is ultrasound imaging [28]. The identification of developmental defects such as skeletal dysplasias is based primarily on decreased dimensions of long bones in relation to gestational age, as well as abnormalities of bone morphology or mineralization. Ultrasound may reveal absence of distal bones in the upper or lower limbs, a defect known as hemimelia, which may be isolated to one bone, but more frequently accompanies other congenital abnormalities, i.e. syndactyly, polydactyly. EEC syndrome is characterized by the following triad of defects: ectrodactyly, ectodermal dysplasia and cleft lip and palate, as well as pes valgus. Congenital absence of the tibia is characterized by a shortening of the lower limbs, with a supinated foot and unstable knee joint [25–27]. Hemimelia of the tibia can be detected ultrasonically as early as at 16.5 weeks of pregnancy [25].

Radiography [4], ultrasound imaging [18], CT [3] and MRI [29] may considerably improve the prenatal diagnosis of congenital defects. Victoria et al. [30] and Cassart et al. [31] demonstrated the advantage of 3D CT in comparison with 2D ultrasound in the diagnostics of skeletal dysplasias. A currently limiting factor for CT examinations is a lack of numerical data describing the fetal skeletal system at particular gestational weeks in comparison with ultrasound examinations. Currently, MRI is becoming the most accurate diagnostic tool used to assess fetal anatomy, both in in utero fetuses and autopsied fetuses. MRI in fetal anatomy examinations is critically essential in the 2nd and 3rd trimesters of pregnancy, when ultrasound imaging offers either ambiguous or limited findings, exemplified by oligohydramnios or breech presentation of the fetus [29]. Due to advances in fetal surgery, the use of MRI mainly refers to congenital defects of the central nervous and skeletal systems, as well as congenital defects of thoracic and abdominal organs [32]. The newly developed cine-MRI techniques provide an innovative insight into the movements of the entire fetus in the three-dimensional environment of the uterus during pregnancy [33].

## Conclusions

1. The size of tibial shaft ossification displays neither sex nor laterality differences.
2. Tibial shaft ossification follows quadratic functions with respect to its length, projection surface area and volume, and linear functions with respect to its proximal, middle and distal transverse diameters.
3. The obtained morphometric data of tibial shaft ossification are considered normative age-specific references of relevance in both the estimation of fetal ages and the ultrasound diagnostics of congenital defects.
